# Bradykinin-Mediated Angioedema Induced by Drugs

**DOI:** 10.3390/jcm14165712

**Published:** 2025-08-12

**Authors:** Chiara Suffritti, Samantha Chan, Anne Lise Ferrara, Eralda Lekli, Francesco Palestra, Gülseren Tuncay, Stefania Loffredo, Maria Bova

**Affiliations:** 1 Fondazione IRCCS Ca’ Granda Ospedale Maggiore Policlinico, Angelo Bianchi Bonomi Hemophilia and Thrombosis Center, 20122 Milan, Italy; 2 Department of Clinical Immunology & Allergy, Royal Melbourne Hospital, Parkville, VIC 3052, Australia; 3 Department of Infectious Diseases & Immunology, Austin Hospital, Heidelberg, VIC 3084, Australia; 4 Immunology Division, Walter and Eliza Hall Institute of Medical Research (WEHI), Parkville, VIC 3052, Australia; 5 Department of Translational Medical Sciences, University of Naples Federico II, 80131 Naples, Italy; anneliseferrara@gmail.com (A.L.F.); f.palestra97@gmail.com (F.P.); stefanialoffredo@hotmail.com (S.L.); 6 WAO Center of Excellence, 80131 Naples, Italy; 7 Faculty of Medicine, University of Medicine, 1005 Tirana, Albania; leklieralda@yahoo.com; 8 Institute of Allergology, Charité—Universitätsmedizin Berlin, 13353 Berlin, Germany; seren_tuncay@hotmail.com; 9 Fraunhofer Institute for Translational Medicine and Pharmacology ITMP, Immunology and Allergology, 12203 Berlin, Germany; 10 Division of Allergy and Clinical Immunology, Faculty of Medicine, Hacettepe University, Ankara 06100, Türkiye; 11 Italian Network for Hereditary and Acquired Angioedema (ITACA), 20125 Milan, Italy; 12 Center for Basic and Clinical Immunology Research (CISI), 80131 Naples, Italy; 13 Division of Internal Medicine 2, Department of Medicine and Medical Specialties, A. Cardarelli Hospital, 80131 Naples, Italy; bovamaria@virgilio.it

**Keywords:** angioedema, bradykinin, angiotensin converting enzyme-inhibitors, angiotensin II receptor blockers, gliptins, neprilysin inhibitors, alteplase, tenecteplase, reteplase

## Abstract

Angiotensin-converting enzyme inhibitors (ACEIs) and angiotensin II receptor blockers (ARBs) are among the most widespread drugs for the prevention of cardiovascular mortality and morbidity. Nevertheless, they are known to cause bradykinin (BK)-mediated angioedema (AE), a paroxysmal, localized, self-limiting, and potentially fatal swelling of the subcutaneous and/or submucosal tissue, due to a temporary increase in vascular permeability. Unlike hereditary angioedema (HAE), which can be mediated similarly by BK, no diagnostic tools, guidelines, or drugs have yet been approved for the diagnosis and treatment of acute non-allergic drug-induced AE. Besides ACEIs and ARBs, inhibitors of dipeptidyl peptidase-IV, neprilysin inhibitors, and tissue plasminogen activators are known to cause AE as an adverse effect. Currently, there are insufficient data on the prevention of AE caused by pharmacological therapies. In addition, the molecular mechanisms underlying BK-mediated AE caused by drugs, which are discussed here, are not fully explained. Specific approved drugs and a structured diagnostic workflow are unmet needs and are required for the management of this kind of AE. The aim of this review is to provide physicians with accurate knowledge of potentially life-threatening drug reactions so that they can be better understood and managed.

## 1. Introduction

According to the recent DANCE consensus, angioedema (AE) can be defined as “a paroxysmal, localized, and self-limiting swelling of the subcutaneous and/or submucosal tissue, due to a temporary increase in vascular permeability” [1]. The DANCE expert panel proposed to divide AE into the following types: mast cell-mediated AE (AE-MC), bradykinin-mediated AE (AE-BK), vascular-endothelium dysfunction AE (AE-VE), drug-induced AE (AE-DI), and AE with unknown etiology (AE-UNK) [1,2,3]. AE-BK can be hereditary or acquired. The most common form of AE-BK is hereditary angioedema (HAE) caused by the deficiency of C1 esterase inhibitor (HAE-C1INH), but HAE can also occur with normal plasma levels of C1INH and mutations in genes involved in contact system activation [4]. AE-BK also includes acquired C1INH deficiency with low C1INH (AAE-C1INH) [5,6]. The category of AE-DI includes angiotensin-converting enzyme inhibitors (ACEIs)-induced AE (AE-ACEI) [7]. When ACEIs block the action of ACE, BK is not catabolized into inactive metabolites. Consequently, the increase in the vasoactive peptide BK levels causes vasodilation and enhanced vascular permeability, which leads to mucosal swelling. In addition, multiple drugs like angiotensin II receptor blockers (ARBs), gliptins, neprilysin inhibitors, and tissue plasminogen activators are thought to potentially induce AE mediated by BK, as we will discuss in this review.

The majority of AEs are allergic and mast cell (MC)-mediated. Although this review focuses on BK-mediated AE, it is important to emphasize that several drugs, such as nonsteroidal anti-inflammatory drugs (NSAIDs), antibiotics (particularly beta-lactams), and contrast media, may induce MC-mediated AE. These forms of AE typically are successfully treated with antihistamines, corticosteroids, and epinephrine. Distinguishing BK-mediated AE is critically important for therapeutic decision-making (especially in Emergency Departments, EDs), as such cases are typically unresponsive to standard treatments but may benefit from BK-targeted therapies developed for HAE-C1INH. To date, the evaluation of contact system activation and BK generation has presented methodological issues related to the easy activation of the contact system during collection and handling of samples as well as to the short half-life of BK [8,9]. Consequently, finding a reliable marker to identify BK-mediated AE represents a major need that has yet to be met, and genotyping is required for the diagnosis of HAE if C1INH is not deficient [10,11].

A review of drug-induced AE is extremely important for clinicians who are unfamiliar with potentially severe BK-mediated adverse drug reactions since they include AE involving the floor of the mouth, the base of the tongue, and/or upper airways and can be life-threatening. Depending on the degree of severity, all forms of AE may require emergency treatment. Due to the higher incidence of MC-mediated AE, all acute AEs are primarily treated with antihistamines and steroids. Since BK-mediated AE is uncommon, usually there are no protocols in place in the EDs, and there is a lack of immediate access to appropriate drugs. A survey of British EDs demonstrated that medications required to treat BK-mediated AE were available in the majority of hospitals with access to specialist immunology services but were not accessible in the ED, and only half of the hospitals surveyed had established guidelines for the use of these medications [12]. In addition, in contrast with the well-known possibilities of therapy for HAE-C1INH, no drug has been approved to date for the treatment of AE-ACEI. Awareness of BK-induced AE and the therapeutic options for treating it will improve outcomes in the EDs [13,14].

The rationale behind a review on drug-induced AE is to provide physicians with accurate knowledge so that potentially life-threatening drug reactions can be better understood and managed. In addition, there is an unmet need to effectively communicate to patients the risk of AE associated with the use of some medications. As reported in a study carried out to assess patients’ level of awareness of adverse effects of ACEIs, patients’ knowledge of adverse effects of these drugs was poor [15,16]. To optimize patient care and safety, open communication between physician and patient is essential.

In order to prepare this review, we conducted a PubMed literature search up to May 2025.

## 2. Angiotensin-Converting Enzyme Inhibitors-Induced Angioedema

ACEIs are one of the most common causes of AE-DI [17]. ACEIs are medications used in the treatment of cardiovascular and renal diseases, including heart failure, acute coronary syndrome, nephrotic syndrome, diabetes, and hypertension. The incidence of AE-ACEI is thought to be 0.1–0.7%, but in some studies, the reported rate is as high as 2.2 to 6% [17,18,19]. In 2011, ACEIs were taken in 164 million prescriptions in the USA [20]. Over the past years, ACEI consumption has increased to more than 40 million people worldwide, so the prevalence of AE-ACEI is likely to increase [21,22]. Among adverse effects of ACEI use, AE is the major cause of hospitalization [23,24]. The severity of AE may be up to 20% life-threatening, affecting the larynx and upper respiratory tract [22,25]. There is no clear rate, but fatal cases have been reported in the literature [26]. While belonging to the African American race, smoking, older age, female gender, heart failure, previous history of drug hypersensitivity, surgery, trauma, immunosuppression in cardiac and renal transplant recipients, and polymorphisms of genes related to BK metabolism were stated as risk factors (Table 1), diabetes mellitus and obesity were found to be protective factors [27,28]. Regarding genetic risk loci, recent genome-wide association studies (GWASs) identified two loci with a genome-wide significant association with AE-ACEI: the BK receptor B2 (*BDKRB2*) locus on chromosome 14 [29] and the *KCNMA1* (calcium-activated potassium channel subunit alpha-1) locus on chromosome 10 [30]. In addition, common variations in *KCNMA1* have been reported to be associated with the risk of AE induced by ARB treatment [30]. Genetic variants in *XPNPEP2*, which encodes a BK-metabolizing enzyme, are associated with AE-ACEI in three case-control candidate gene studies [31]. Furthermore, an exome sequencing study revealed associations between ACEIs/ARBs-induced AE and the common factor V Leiden mutation, as well as other rare variants in the *F5* (coagulation factor V) gene [32]. A recent GWAS meta-analysis [33] identified three genome-wide significant risk loci: one of these, located on chromosome 20q11.22, has not been implicated previously in AE-ACEI. Other analyses in this study highlighted previously reported genes *BDKRB2* and *F5* as well as novel candidate genes, *PROCR* (which encodes the protein C receptor, EPCR) and *EDEM2* (endoplasmic reticulum degradation enhancing alpha-mannosidase-like protein 2). Both EPCR and *EDEM2* influence the levels of protein C. Despite these advances, the precise underlying genetic causes of ACEIs/ARBs-induced AE remain largely unclear.

**Figure 1:**
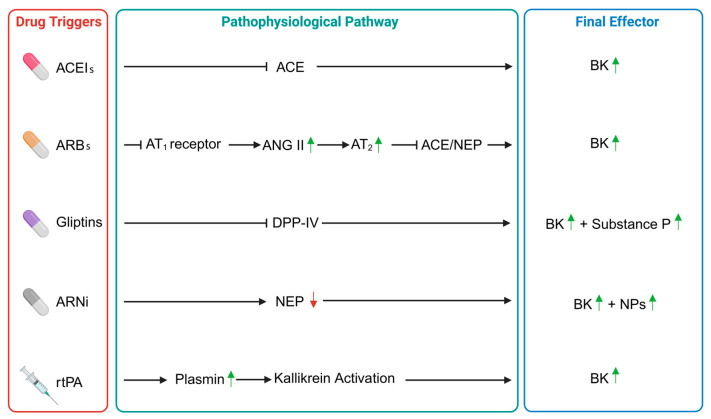
Pathophysiological pathways of different bradykinin-mediated angioedema induced by drugs. ACEIs: ACE inhibitors, ACE: angiotensin-converting enzyme, BK: bradykinin, ARBs: angiotensin II receptor blockers, AT_1_: angiotensin II type 1 receptor, ANG: angiotensin, NEP: neutral endopeptidase, DDP-IV: dipeptidyl peptidase-IV, ARNi: angiotensin receptor/neprilysin inhibitor, NPs: natriuretic peptides, rtPA: recombinant tissue plasminogen activator. Green arrows indicate an increase, the red arrow indicates a decrease.

The pathophysiology of AE-ACEI is not yet clearly understood [34]. However, it is known that BK plays a main role (Figure 1). BK is a product of the kinin-kallikrein system, and it was discovered in 1948 when detected in animal plasma after injecting venom from *Bothrops jararaca*, the South American pit viper [20]. BK is a potent vasodilator and increases vascular permeability and non-vascular smooth muscle contraction [20]. High doses of BK bind to vascular B2 receptors, causing a rise of cGMP and NO; consequently, vascular permeability increases and fluid accumulation in the interstitial space occurs [27,28]. The inactivation of BK and the conversion of angiotensin (Ang) I to angiotensin II in the lungs were found to be catalyzed by the same enzyme, ACE [35]. The discoveries of BK and ACE in plasma were stepping stones in the pursuit of drugs affecting the renin-angiotensin-aldosterone system (RAAS) [36]. ACEIs cause BK accumulation, slow processing of C-terminal arginine residues of BK, and prolong the biological activity of BK metabolism [27]. According to one study, during an acute episode of AE with ACEIs, BK levels can increase up to seven-fold [9]. Furthermore, in the study of Carucci et al., it was reported that ACEI-induced AE patients were prone to increased vascular permeability with high baseline Vascular Endothelial Growth Factor (VEGF)-A, VEGF-C, and secreted phospholipase A_2_ (sPLA_2_) levels and that this elevation could play an important role in the decrease in BK catabolism and increase in BK levels [17]. Blais et al. showed that in ACEI-induced AE, 50% of patients had an enzyme defect in the metabolism of des-Arg-9-BK, which is an active metabolite of BK [37]. In addition, Molinaro et al. [38] reported an abnormality of endogenous des-Arg-9-BK degradation in plasma of patients with ACEI-associated AE, suggesting a pathogenetic mechanism in the catabolic site of kinin metabolism.

When clinicians encounter a recurrent form of nonspecific AE with normal C1INH levels, diagnostic biomarkers that distinguish hereditary and acquired AE are insufficient [15]. In HAE-C1INH patients, laboratory testing shows abnormal levels of C1INH and high levels of BK [39]. C4 and D-dimer levels can also be monitored if an acute attack is suspected. However, Bas et al. found increased C-reactive protein (CRP) levels in a retrospective cohort study of 25 patients with AE-ACEI [40]. At the symptomatic stage, all patients with AE-ACEI had significantly increased CRP plasma levels and fibrinogen in comparison to normal values found in patients with AE of unknown cause [41]. The clinical symptoms of AE-ACEI are similar to those observed in patients with C1INH deficiency, mainly affecting the face, oral mucosa, tongue, lips, pharynx, and larynx, being predominantly asymmetric, and being potentially life-threatening when laryngeal edema develops [27,41,42]. Furthermore, AE of the small intestine is a rare adverse reaction of ACEIs [43,44]. It is important to recognize this side effect, as the drug is one of the most prescribed medications. As the symptoms of visceral AE may mimic other diseases, it may lead to misdiagnosis or unnecessary workup and procedures.

The onset of AE, while most often occurring within the first month of treatment, frequently occurs after significant exposure [45]. Although two-thirds of the cases experience AE within the first 90 days of ACEI intake, it has been shown that, less frequently, the first episode can occur up to 10 years after drug initiation [18,25,46]. Of note, multiple medications may cause AE, including aspirin and other NSAIDs, and these should be questioned in the patient’s history [18,47,48]. AE-ACEI can resolve spontaneously in the acute phase. Discontinuation of the drug is recommended and useful to prevent recurrence of symptoms. Symptoms of AE secondary to ACEIs usually tend to resolve within 24–48 h of discontinuing the culprit drug. Treatment of AE is support for the patient’s airway as required or simply drug discontinuation in mild cases [28]. Some cases of late-onset AE may appear even weeks after the discontinuation of the ACEIs [49,50]. This has been widely shown in a follow-up study of patients in whom 89% of cases had no further events after stopping the medication [51]. There are currently no FDA-approved medications for AE-ACEI; however, several therapies have been reported to be effective with variable efficacy, including fresh frozen plasma (FFP) (which contains kininase II), ecallantide (a direct inhibitor of plasma kallikrein), icatibant, tranexamic acid (TXA), and C1INH concentrate [52,53,54]. Antihistamines, steroids, and epinephrine are effective in MC-mediated AE but not in BK-mediated types (which include AE-ACEI, AAE-C1INH, and HAE) [55]. In cases of severe AE, patients should be monitored for airway compromise and fatal complications because of the risk of relapses, despite withdrawal of the offending drug [27]. In previous case studies, the use of icatibant shortened the resolution of AE compared with classic drugs [54,55,56]. In addition, B2 receptor antagonists are used (off-label) for AE-ACEI in France but potentially have limited efficacy in Black patients [57]. A meta-analysis that included three randomized controlled trials (RCTs) evaluated the effectiveness of icatibant therapy for AE-ACEI, but the benefit of icatibant therapy over placebo or conventional treatment strategies could not be shown [58]. FFP has also been shown to be useful for the treatment of AE-ACEI [42,57,58,59,60,61,62]. The mechanism involved in the therapeutic effect of FFP is the presence of angiotensin II in plasma, which catalyzes the degradation of excess BK. However, FFP can also contain complement components that can make the AE more severe and long-lasting. Thus, it is not recommended during an acute attack [63]. It is important to note that ACEIs should be avoided in BK-associated AE. ACEIs are trigger factors for some patients with HAE with normal C1INH [64]. These diseases have low penetrance, and in some cases the symptoms appear just after the intake of ACEIs.

Hypertension in children and adolescents remains a significant health care concern, ranging from 3% in the general population to up to 25% in obese children. The use of antihypertensive medications in this young population is an emerging public health concern and will most likely continue to increase [65]. The adverse effects of ACEIs in pediatric patients were only evaluated in three studies [66,67,68], and there are few reported cases in children [69,70,71,72,73]. In general, ACEIs (with enalapril being the most common) induced a non-histaminergic AE mostly involving the face, tongue, and lips together with the neck and oropharynx.

When AE-ACEI has been diagnosed, treatment with ACEIs should be strictly avoided, and an alternative drug should be prescribed. A follow-up study in a large group of AE-ACEI patients observed for 14 years showed that the interruption of ACEIs and the switch to another antihypertensive drug prevented recurrences of AE in about 50% of patients [74].

## 3. Angiotensin II Receptor Blockers-Induced Angioedema

ARBs, also known as sartans, are a cornerstone of antihypertensive therapy and are widely used due to their efficacy and favorable tolerability profile. Unlike ACEIs, ARBs do not inhibit BK breakdown directly and historically were believed to carry little to no risk of inducing BK-mediated AE. However, emerging evidence has refined this view. Even if the incidence of ARBs-associated AE is significantly lower than that associated with ACEIs, the risk is not negligible, particularly in certain patient populations. New clinical, mechanistic, and pharmacogenetic insights over the past several years support a more nuanced understanding of this adverse drug reaction.

Although ARBs do not inhibit ACE, they may indirectly promote BK accumulation (Figure 1) through complex effects on the RAAS. Specifically, ARBs block the angiotensin II type 1 receptor, leading to increased levels of circulating Ang II, which may in turn stimulate the angiotensin II type 2 receptor (AT2). Activation of AT2 has been shown to downregulate ACE and neutral endopeptidase (NEP), two major enzymes responsible for BK degradation [75,76]. Moreover, AT2 stimulation may sensitize BK B2 receptors, further amplifying vascular responses to BK. These indirect mechanisms offer a plausible explanation of the reason why ARBs, despite not acting directly on BK metabolism, may still lead to AE in susceptible individuals. Ang II blockade also reduces plasma levels of aldosterone and norepinephrine [77].

ARBs have a similar efficacy to ACEIs in reducing hypertension and preventing cardiovascular events but tend to be better tolerated, including lower rates of antihypertensive-associated cough [78,79,80,81,82]. Consequently, ARBs are a widely prescribed medication. Population-based studies and meta-analyses have established that the incidence of AE with ARB use ranges between 0.03% and 0.2%, substantially lower than the incidence with ACEIs [83,84] (Table 1). In a recent analysis of the literature, the risk of AE with ARBs was similar to that with placebo (odds ratio: 1.62; 95% CI: 0.17–15.79) [82]. Moreover, an analysis of two RCTs, two retrospective cohorts, and one meta-analysis estimated the incidence of AE in less than 10% of patients who receive an ARB after experiencing AE-ACEI [85,86]. A pooled analysis of 31 studies in 12,188 patients showed the incidence of AE associated with aliskiren monotherapy, a direct renin inhibitor, was 0.4%, with no serious AE event reported [87]. The mechanism seems to be related to BK increase via the angiotensin II activation of angiotensin II type-2 (AT2) receptors and the subsequent inhibition of BK breakdown [88,89]. The risk of AE is highest during the initial weeks of therapy, with most cases occurring within the first 30 days of drug initiation. In a recent study aimed at investigating non-genetic association factors with ACEIs-/ARBs-induced AE, increased age, smoking, allergies, and a history of previous AE were identified as associated factors for ACEIs-/ARBs-induced AE. In most patients, the swelling affected the face, lips, and tongue [90].

Analysis of arterial blood in individuals on losartan has found that BK levels are increased to a level similar to individuals on ACEIs, a rise presumably mediated via the effects of ARBs on metabolism of BK by ACE and neutral endopeptidase [75,76]. This mechanism is thought to be contributing to the pathogenesis of ARBs-associated AE [83]. Interestingly, multiple trials have indicated that ARBs do not significantly increase the likelihood of AE compared to placebo [91,92,93].

Some pharmacovigilance data suggest that individual ARBs may differ in their risk of inducing AE. A recent analysis using the FDA Adverse Event Reporting System (FAERS) database found stronger safety signals for losartan and irbesartan compared to other ARBs, while valsartan appeared to be associated with a relatively lower risk. However, these findings need confirmation in prospective epidemiological studies, as current evidence remains limited and inconclusive regarding molecule-specific risk [94].

Pharmacogenetic studies have further elucidated the polygenic basis for susceptibility to RAAS inhibitor-induced AE. A GWAS study by Rasmussen et al. identified variants in the *KCNMA1* gene as significantly associated with AE induced by both ACEIs and ARBs [30]. *KCNMA1* encodes a calcium-activated potassium channel involved in vascular tone regulation, potentially modulating the endothelial response to BK. More recently, a large multi-ancestry meta-GWAS involving over 1000 patients with ACEIs- or ARBs-associated AE identified three genome-wide significant risk loci, including novel associations at the *PROCR* and *EDEM2* loci [33]. These genes implicate endothelial protein C receptor signalling and the endoplasmic reticulum degradation pathway in disease pathogenesis, suggesting an intersection between coagulation, endothelial integrity, and BK-mediated vascular permeability.

A commonly encountered clinical scenario involves determining whether a patient with a history of AE-ACEI can be safely prescribed an ARB. It was previously believed that the risk of AE recurrence after switching from an ACEI to an ARB could be as high as 10%, and strong caution was advised when initiating such a treatment trial [84,87,95,96]. However, this risk has likely been overestimated due to the potential of ACEIs to cause AE weeks to months after drug cessation [74], as well as potential overdiagnosis of BK-mediated AE in individuals on an ACEI or ARB [97]. There is strong evidence, including nationwide registry studies, that supports the safety of ARB use in individuals with prior AE-ACEI [83,98,99]. It is reasonable to consider an ARB in such patients, particularly when cardiovascular benefits are expected to be significant, provided that there is a sufficient washout period and close clinical monitoring. A careful assessment of risk factors, patient education on symptom recognition, and close monitoring during the initial weeks of therapy are recommended.

## 4. Angioedema in DPP-IV Inhibitors

Gliptins are inhibitors of multifunctional protein dipeptidyl peptidase-IV (DPP-IV) or cluster of differentiation 26 (CD26), which represents a proteolytic enzyme, receptor, and costimulatory protein. It is involved in adhesion, apoptosis, and immune response [100], and it is expressed in various organs and cells [101], including vascular endothelial cells [102]. DPP-IV participates in various physiological and pathological processes by regulating energy metabolism, inflammation, and immune function. DPP-IV inhibitors are approved for the treatment of type 2 diabetes mellitus [101].

Based on post-marketing surveillance, AE has been reported as rare (≥1/10,000 to <1/1000) for linagliptin [103], alogliptin [104], and vildagliptin [105] (Table 1) and as an adverse reaction of not known frequency (cannot be estimated from available data) for saxagliptin [106] and sitagliptin [107], but can be underreported. Analysis of 29,163,222 reports identified 588 cases of DPP-IV inhibitor-associated AE. Significant safety signals have been detected for DPP-IV inhibitor monotherapies [108].

The exact mechanism of AE in DPP-IV inhibitor therapy is likely multifactorial, involving BK accumulation and immune system activation, among other factors (Figure 1). DPP-IV is involved in the breakdown of various bioactive peptides, such as BK and substance P, not only incretin hormones, like gastric inhibitory polypeptide and glucagon-like peptide-1, which stimulate insulin secretion [109]. The level of BK and substance P may increase and lead to a significantly higher risk of AE, as aminopeptidase P, neutral endopeptidase, and carboxypeptidase N may be insufficient for their degradation in predisposed individuals [110,111,112]. While the direct link between these peptides and AE is not fully understood, some of them could theoretically contribute to vascular leakage and to the development of AE.

The inhibition of DPP-IV can lead to the accumulation of other peptides, like B-type natriuretic peptide and neuropeptide Y, which may have vasoactive effects, including increased permeability of the microvasculature in the skin of murine models [113,114], and substance P contributes to tracheal AE through binding of the neurokinin 1 receptor [109,115].

Concomitant use of ACEIs and DPP-IV inhibitors may increase the risk of AE, as the role of DPP-IV and other enzymes involved in the degradation of BK and substance P becomes critical [116,117,118,119]. The theoretical risk of AE is heightened due to accumulation of vasoactive kinins [120]. Significant safety signals for AE with specific DPP-IV inhibitor combinations with RAAS-interfering drugs suggest potential drug-drug interactions [108]. DPP-IV plays a minor role in the degradation of vasoactive peptides when ACE is present and fully functional [109]. Several cases reporting AE induced by gliptins without ACEI concomitance are published [119,121]. DPP-IV inhibitors are suggested to inhibit ACE for a longer duration and may be more likely to cause AE than shorter-acting agents [122]. Linagliptin was the only DPP-IV inhibitor associated with AE regardless of age and the absence of concomitant ACEI use in females in a recent study [108]. Reduced DPP-IV activity may itself predispose individuals to AE [101,109].

Previous history of drug-induced AE, whether from ACEIs or DPP-IV inhibitors, may potentially predict an increased risk of recurrence [111,119,120,122]. Gliptin-induced AE has been occasionally reported in patients who were already receiving concurrent ACEI or had a prior history of ACEI use [110,120,123,124,125].

While AE is rare with DPP-IV inhibitors, certain individual factors—particularly those affecting BK metabolism or immune response—increase the risk. A history of AE, renal dysfunction, and genetic predisposition are among the most important risk factors to consider. Close monitoring for signs of AE is advised, especially during the early phases of treatment.

## 5. Neprilysin Inhibitors-Induced Angioedema

Neprilysin inhibitors target CD10 (membrane metalloendopeptidase), a type II transmembrane glycoprotein and neutral endopeptidase that cleaves peptides at hydrophobic residues [126]. In this way, it affects physiological and developmental functions across tissues by breaking down peptides such as substance P, endothelin, natriuretic peptides, somatostatin, adrenomedullin, glucagon, angiotensin I and II, encephalins [127,128], neurotensin, oxytocin, and BK [126]. CD10 is expressed in various tissues, including hematopoietic tissue [128,129,130], intestines, breasts, kidneys, prostate, lungs, liver, placenta, brain, gonads, adrenal glands, and neurons [131,132,133].

Inhibiting CD10 can enhance the effects of naturally occurring natriuretic peptides (like atrial natriuretic peptide (ANP), brain natriuretic peptide (BNP), and C-type natriuretic peptide (CNP)), which help promote natriuresis, induce vasodilation, and decrease cardiac hypertrophy and fibrosis in heart failure patients [134]. AE was reported as an uncommon adverse event (≥1/1000 to <1/100) (Table 1), affecting 0.5% of patients treated with the combination of neprilysin inhibitors/angiotensin II inhibitor blockers (sacubitril/valsartan) [135]. Despite the current short availability and still limited use of sacubitril/valsartan, life-threatening AE has already been described for this combination [136]. The ACE and the neutral endopeptidase (NEP, also neprilysin) are the most important BK-degrading proteases [137]. In neprilysin inhibitor-induced AE, the elevation of ANP, BNP, and CNP due to neprilysin inhibition, combined with increased BK levels, contributes to vascular permeability and fluid accumulation, leading to AE (Figure 1). The increased concentrations of natriuretic peptides can exacerbate the condition by promoting vasodilation and enhancing fluid leakage into tissues. BK mediates dilatation in the resistance vessels, which leads to hyperfiltration and thus edema formation [123,138,139]. Increased levels of substance P also promote inflammation and vascular permeability, adding to the risk of AE. Due to the potential risk of AE when used concomitantly with an ACEI, sacubitril/valsartan must not be started for at least 36 h after discontinuing ACEI therapy [135].

A recent study did not identify an increased risk of AE among sacubitril/valsartan new users compared with ACEI or ARB users, but there was an increased risk of AE among sacubitril/valsartan users who recently switched from ACEI or ARB compared with sacubitril/valsartan new users [140]. A common denominator is that there is no approved therapy for BK-mediated AE as a drug side effect [139].

## 6. Recombinant Tissue Plasminogen Activator-Induced Angioedema

Alteplase is a thrombolytic agent widely used for the treatment of acute ischemic strokes. It is the recombinant form of tissue plasminogen activator (rtPA), an enzyme that catalyzes the conversion of plasminogen to plasmin, resulting in fibrinolysis [141,142].

Bleeding is one of the major risks for alteplase therapy, but it should not be neglected that in some clinical cases (0.2 to 7.9%) alteplase-induced AE can occur [143,144,145,146,147] (Table 1). It manifests usually as a transient swelling of the tongue, lips, and tissue of the oropharynx during or shortly after alteplase administration [148]. It is often contralateral to the ischemic hemisphere and usually resolves within 24 h [149]. Available data suggest that female sex, Caucasian race, hypertension, diabetes, dyslipidemia, and ACEI treatment may increase the risk of oral AE occurrence following the administration of alteplase [150,151,152]. AE seems to occur more frequently after use for ischemic stroke than for other indications, such as myocardial infarction or pulmonary thromboembolism [150]. A potential pathogenic pathway involved in alteplase-induced AE development has been hypothesized. Alteplase converts plasminogen to plasmin, resulting in fibrinolysis; plasminogen activation, in addition to fibrinolysis, may activate the kinin system [153], leading to BK accumulation (Figure 1). Moreover, prostaglandins (mostly prostaglandin D2 or PGD2), histamine, chymase, tryptase, and leukotrienes (LTB4, LTC4, LTD4, and LTE4) can all be released as a result of MC degranulation triggered by plasmin activation. BK and MC degranulation mediators can increase vascular permeability and induce fluid extravasation, leading to the development of AE [151]. In BK-mediated AE (such as AE-ACEI), treatment with antihistamines, corticosteroids, and epinephrine is generally ineffective [142]. This pathogenic pathway could justify many case reports describing patients with alteplase-induced AE treated with the administration of icatibant, a synthetic BK B2 receptor antagonist [154,155,156,157,158,159]. Other case reports mention the use of FFP and C1INH as treatment options for AE secondary to administration of alteplase [144,160]. In the published reports describing treatment of alteplase-induced AE with drugs developed to treat BK-mediated AE (namely icatibant, FFP, and C1INH), these medications are used as a second option after the classical anti-allergic drugs. This approach and the lack of clinical trials make it difficult to establish the efficacy of these therapies. In spite of this, in the latest guidelines for the management of patients with acute ischemic stroke, the authors propose icatibant or C1INH as alternatives to manage orolingual AE associated with endovenous alteplase administration. They do not clarify when it is convenient to administer these drugs and propose them by describing their efficacy in HAE and AE-ACEI. There is a clear need for more data on this topic in order to create a flowchart available for neurologists and resuscitators to deal with a potential life-threatening adverse event of alteplase [161].

Recent studies have shown that tenecteplase (TNK), an engineered variant of alteplase, delivers clinical benefits similar to those of alteplase. Some guidelines have recommended either alteplase or TNK for patients with acute ischemic stroke within 4.5 h after known onset [162]. TNK is a genetically modified tPA with higher fibrin specificity, a longer half-life, and reduced systemic coagulopathy [163]. In 2023, Rose et al. published a systematic review and meta-analysis comparing complications of intravenous TNK versus alteplase for the treatment of acute ischemic stroke [164]. The main described complications were intracranial and extracranial hemorrhage and AE. Within the RCTs included in this paper, AE was documented in 0.56% of patients treated with TNK and 0.63% of patients treated with alteplase. Within the largest clinical trial [165], 9 of 800 (1.1%) patients in the TNK arm and 9 of 763 (1.2%) in the alteplase arm experienced AE. Xiang et al., in 2025, conducted a large-scale retrospective pharmacovigilance study using the FAERS database [166]. Their study suggested that alteplase use is associated with the occurrence of AE among stroke patients, while current evidence does not support an association between use of TNK and AE. They suggest considering TNK as an alternative treatment to alteplase, particularly for those patients with identified risk factors, if available. Sekita and colleagues retrospectively analyzed their stroke registry to compare clinical and procedural data from acute ischemic stroke patients treated with alteplase and those treated with TNK. During a twelve-month period, 276 patients underwent intravenous thrombolysis. No significant differences were observed in safety outcomes, including intracranial hemorrhage, symptomatic intracranial hemorrhage, or AE (3% with TNK vs. 1% with alteplase; *p* = 0.18) [167]. Due to these controversial findings, it is clear that it is fundamental to collect additional data to clarify the risk of TNK inducing AE.

Reteplase is a recombinant plasminogen activator, approved for the treatment of acute myocardial infarction in many geographic regions. Li et al. demonstrated that, in patients with acute ischemic stroke, reteplase was superior to alteplase with respect to an excellent functional outcome at 90 days [162]. However, patients receiving reteplase had a higher incidence of any intracranial hemorrhage than those receiving alteplase. In this paper the authors do not describe cases of AE related to administration of reteplase; in spite of this they describe the occurrence of “skin and subcutaneous tissue disorders” in 5.6% of patients, without further details.

## 7. Discussion

According to the recent DANCE consensus, the new category of AE-DI was created to include all drug-induced AE [1]. Consequently, the category of AE-DI comprises not only AE due to ACEIs, ARBs, DPP-IV inhibitors, neprilysin inhibitors, and tissue plasminogen activators, but also AE caused by NSAIDs. NSAIDs may trigger AE via eicosanoid pathways or via MC degranulation. For that reason, including AE triggered by different mechanisms in the same category could be misleading.

AE is a rare complication of some medications and, when affecting the upper airways, is a challenge for physicians in the EDs since MC- or BK-induced forms of AE cannot be readily distinguished. The diagnosis of BK-mediated induced AE is particularly difficult due to the absence of specific biomarkers that can be rapidly measured. As a result, it remains a diagnosis of exclusion. These forms of AE frequently involve the face and airways of patients who are diagnosed in emergency settings, where the time-sensitive nature of care makes it especially difficult to promptly rule out alternative diagnoses.

It is important to emphasise that ACEIs can trigger AE in patients affected by HAE with normal C1INH [168], while a few patients have been reported to have received ACEIs without any influence on their disease [169]. ACEIs, however, should be avoided in BK-associated AE. Regarding the treatment of ACEI-AE, three randomized studies evaluated the efficacy and safety of icatibant in AE-ACEI. The first study, a randomized double-blind phase II study, included 27 patients with AE-ACEI [170]. The primary endpoint of the study was time to resolution of symptoms, and patients were treated approximately 6 h after the onset of symptoms. This trial provided the first proof of the efficacy of icatibant in ACEI-induced AE. In the other two studies [171,172], which enrolled 31 and 121 patients with ACEI-induced AE, respectively, the “standard of care” therapy (glucocorticoids and H_1_ antihistamines) was given to about 90% of patients in both study arms. In addition, treatment was administered in the two trials on average 10.3 and 7.8 h after the onset of symptoms. In the latter two studies, no significant difference regarding time to discharge was found between placebo and icatibant. These discrepancies may be due to heterogeneity in study design, such as differences in inclusion criteria, population characteristics, and, crucially, the timing of drug administration. In some trials, icatibant was administered more than 7–10 h after symptom onset, a delay that could significantly reduce its therapeutic efficacy. Early intervention is likely critical in achieving clinical benefit with BK-targeted therapies. Consequently, a final assessment of the efficacy of icatibant in ACEI-induced AE cannot be made.

The proposed pathogenetic mechanism underlying alteplase-induced AE involves the activation of plasminogen to plasmin, which may, in turn, stimulate the kallikrein–kinin system, leading to an accumulation of BK. Additionally, plasmin activation can induce MC degranulation, resulting in the release of mediators that, together with BK, contribute to increased vascular permeability. However, the precise mechanisms responsible for alteplase-induced AE remain unclear and warrant further investigation. With regard to treatment of this form of AE, published case reports indicated that therapies such as icatibant, FFP, and C1INH were typically employed only after failure of standard anti-allergic medications. In addition, the lack of clinical trials complicates the assessment of the efficacy of these therapies.

Although AE is a recognized complication of TNK administration, detailed descriptions of individual clinical cases are scarce. Two case reports illustrated the use of C1INH in TNK-associated orolingual AE [173,174], whereas no reports describing the use of icatibant in this context could be identified. Similarly, the description of cases of reteplase-induced AE is lacking in the literature.

## 8. Conclusions

BK-mediated AE induced by drugs is a rare but underestimated complication. ACEIs, ARBs, inhibitors of dipeptidyl peptidase-IV, neprilysin inhibitors, and tissue plasminogen activators are known to cause AE as an adverse effect. Its frequency is variable depending on the class of drug considered. Risk factors also vary and include gene mutations, smoking, and older age. BK-mediated AE induced by drugs is still difficult to diagnose, and only off-label treatment exists. Specific approved drugs and a structured diagnostic workflow are required for the management of this kind of AE.

## Figures and Tables

**Table 1:** The most common agents of AE-DI.

	ACEIs	ARBs	DPP-IV Inhibitors	Neprilysin Inhibitors	Alteplase
**Drug class**	Antihypertensive	Antihypertensive	Gliptins	Antihypertensive	Thrombolytic agent
**Mechanism of Action**	Inhibition of ACE	Selective blocking of angiotensin II	Inhibition of DPP-IV	Inhibition of NEP (neutral endopeptidase)	Conversion of plasminogen in plasmin
**Reported frequency**	0.1–6%	0.03–0.2%	≥1/10,000– <1/1000	≥1/1000– <1/100	0.2–7.9%
**Underlying pathomechanisms**	Defect of des-Arg-9-BK	Increased activity of RAAS	Scarce degradation of BK	Scarce degradation of BK	Kinin system activation by plasminogen
**Risk Factors:**					
**A.** **Mutations**	*BDKRB2*				
	*KCNMA1*	*KCNMA1*			
	*XPNPEP2*				
	*F5*	*F5*			
	*20q11.22*				
	*PROCR*	*PROCR*			
	*EDEM2*	*EDEM2*			
**B.** **Other**	African American race		Concomitant use of ACEIs	Switching from ACEIs or ARBs	Caucasian race
	Smoking	Smoking	Renal dysfunction		Female sex
	Older age	Older age	History of drug-induced AE		Hypertension
	Female sex	Allergies			Diabetes
	Heart failure				Dyslipidemia
	History of drug hypersensibility				ACEIs treatment
	Immunosuppression				
**Localization of AE**	Face, oral mucosa, tongue, lips, pharynx, larynx	Face, lips, tongue			Tongue, lips, oropharynx
**Therapy**	ACEIs discontinuation				Fresh frozen plasma
	Fresh frozen plasma				C1INH concentrate
	Ecallantide				Icatibant
	Icatibant				
	Tranexamic acid				
	C1INH concentrate				
